# Prospective Analysis of the Association of a Common Variant of *FTO* (rs9939609) with Adiposity in Children: Results of the IDEFICS Study

**DOI:** 10.1371/journal.pone.0048876

**Published:** 2012-11-14

**Authors:** Fabio Lauria, Alfonso Siani, Karin Bammann, Ronja Foraita, Inge Huybrechts, Licia Iacoviello, Anna C. Koni, Yannis Kourides, Staffan Marild, Denes Molnar, Luis A. Moreno, Iris Pigeot, Yannis P. Pitsiladis, Toomas Veidebaum, Paola Russo, IDEFICS Consortium

**Affiliations:** 1 Unit of Epidemiology and Population Genetics, Institute of Food Sciences, Consiglio Nazionale delle Ricerche, Avellino, Italy; 2 BIPS-Institute for Epidemiology and Prevention Research, Bremen, Germany; 3 Unit Nutrition and Food Safety, Department of Public Health, Faculty of Medicine and Health Sciences, Ghent University, Ghent, Belgium; 4 Fondazione di Ricerca e Cura “Giovanni Paolo II”, Università Cattolica del Sacro Cuore, Campobasso, Italy; 5 Institute for Cardiovascular and Medical Sciences, College of Medical, Veterinary and Life Sciences, University of Glasgow, Glasgow, United Kingdom; 6 Research and Education Foundation of Child Health, Strovolos, Cyprus; 7 Department of Paediatrics, Queen Silvia Children's Hospital, University of Gothenburg, Gothenburg, Sweden; 8 Department of Paediatrics, Medical Faculty, University of Pecs, Pecs, Hungary; 9 Growth, Exercise, Nutrition and Development (GENUD Research Group), University of Zaragoza, Zaragoza, Spain; 10 National Institute for Health Development, Tallinn, Estonia; The Children's Hospital of Philadelphia, United States of America

## Abstract

**Objectives:**

We investigated cross-sectionally and longitudinally the relationship between *FTO* rs9939609 and obesity-related characteristics in the European children of the IDEFICS project and the interaction of this variant with a lifestyle intervention.

**Population and Methods:**

A cohort of 16224 children (2–9 years) was recruited into a population-based survey (T0) from eight European countries. A second survey (T1) reassessed the children two years later. A random sample of 4405 children was extracted for genetic studies. 3168 children were re-examined two years later. Half of them underwent a lifestyle intervention program. The *FTO* rs9939609 was genotyped. Weight, height, waist circumference, triceps and subscapular skinfolds were measured at T0 and T1.

**Results:**

At T0, the risk A allele of rs9939609 was significantly associated with higher values of body mass index (BMI), waist circumference and skinfolds (age, sex, and country-adjusted p-values: all p<0.001) and with a statistically significant increased risk of overweight/obesity.

Over the two year follow-up, no interaction between genotype and intervention was observed. The A allele was associated to a significantly higher increase in all the anthropometric variables examined at T0 independently from the study group (intervention versus control) (p-values: all p<0.002, adjusted for age, sex, country, intervention/control study group, T0 values, and individual time interval between T0 and T1). Over the two-year follow–up, 210 new cases of overweight/obesity occurred. A statistically significant higher incidence of overweight/obesity was associated to the A allele [OR_A_ = 1.95, 95% CI = (1.29; 2.97)].

**Conclusions:**

We confirmed the association between the *FTO* rs9939609 and body mass and overweight/obesity risk in European children. The main finding of the study is that the A allele carriers present higher increase of body mass and central adiposity over time and higher risk of developing overweight/obesity during growth, independently from intervention measures.

## Introduction

Obesity is a complex disease, arising from genetic and environmental influences, and gene x environment interactions [1]–[3]. Several genome-wide association (GWA) studies have been conducted in recent years, linking common genetic variations to obesity and related metabolic traits [4]. Among them, the role of the Fat Mass and Obesity Associated gene (*FTO*) has robustly and consistently been found to associate with common obesity [5]. Almost simultaneously, three well-powered studies published in 2007 identified a cluster of common SNPs in the first intron of *FTO* on chromosome 16 as unexpected but strong contributors to both adult and childhood obesity phenotypes [6]–[8].

Human *FTO* has been long considered “a gene of unknown function in an unknown pathway” [6] and presents high homology with the murine *Fto*, located on mouse chromosome 8 [9]. In recent years, several papers shed light on its physiological role but “a complete understanding of the true cellular function of FTO remains a puzzle”, as recently reviewed by Larder et al [10].

The association between the common rs9939609 polymorphism of *FTO* with body mass index (BMI), other indices of adiposity and obesity risk has been consistently replicated in adult populations [11]. Although rs9939609 is the most studied variant in the *FTO* gene, it is in strong linkage disequilibrium with several other SNPs located in the first intron as well [6]–[8]. It represents thus a convenient surrogate for association studies of the *FTO* gene in European populations, but its causal role remains to be elucidated.

A recent collaborative meta-analysis of 14 genome-wide association studies consisting of 5530 cases (≥95th percentile of BMI) and 8318 controls (<50th percentile of BMI) of European ancestry confirmed that *FTO* gave the strongest evidence for association with elevated adiposity in childhood and adolescence [12].

Recent studies attempted to evaluate the effect of *FTO* rs9939609 on the age-related increase of BMI and of other adiposity indices, using retrospectively collected data and in some cases reported measures of adiposity [13]–[24]. Some of them investigated the influence of this common variant on body mass development from childhood to adulthood [14], [16], [20]–[24].

Evidence concerning the potential modifying effect of the *FTO* gene on body weight changes achieved by lifestyle intervention is relatively limited [25]. In fact, the studies that analyzed the associations between this genotype and the effect of lifestyle intervention programs both in adults [26]–[29] and in children [30]–[32] gave heterogeneous results, probably due to different design and methodological and sampling procedures.

The European IDEFICS (Identification and prevention of dietary- and lifestyle-induced health effects in children and infants) Project was set up to determine the etiology of overweight, obesity and related disorders in children [33], [34] and to develop and evaluate a tailored community-oriented intervention program for primary prevention of obesity [35]. This cohort is the largest pan-European children’s cohort established to date, with data collected at baseline (T0, 2007–2008) and two years later (T1, 2009–2010) in eight European countries using standardized procedures.

The primary aim of the present study is to assess the longitudinal effect of *FTO* rs9939609 on BMI and other adiposity indices in the large European prospective cohort of children participating to the IDEFICS project. Additionally, we tested the interaction between the genotype and the effect of the intervention program on the changes in obesity-related traits over the two-year follow-up.

## Materials and Methods

### Ethics Statement

The study was conducted according to the standards of the Declaration of Helsinki. All applicable institutional and governmental regulations pertaining to the ethical use of human volunteers were followed during this research. Approval by the appropriate ethics committees was obtained by each of the eight participating centres carrying out the fieldwork (Belgium: Ethics Committee, University Hospital, Gent; Cyprus: Cyprus National Bioethics Committee; Estonia: Tallinn Medical Research Ethics Committee; Germany: Ethics Committee, University of Bremen; Hungary: Egészségügyi Tudományos Tanács, Pécs; Italy: Comitato Etico, ASL Avellino; Spain: Comité Ético de Investigación, Clínica de Aragón (CEICA); Sweden: Regional Ethics Review Board, University of Gothenburg). Participants were not subjected to any study procedure before both the children and their parents gave their oral (children) and written (parents) informed consent for examinations, collection of samples, subsequent analysis and storage of personal data and collected samples.

### Participants

IDEFICS is a large European multi-center study of childhood obesity. Details of the general design, instruments and survey characteristics can be found elsewhere [34]. A cohort of 16224 children aged 2–9 years was recruited into a population-based baseline survey from eight European countries ranging from the north to the south and from the east to the west (Belgium, Cyprus, Estonia, Germany, Hungary, Italy, Spain, and Sweden). The baseline survey (T0) was the starting point of the cohort study aimed to prospectively evaluate the role of the factors assessed at baseline on the development of overweight/obesity over time and to assess the feasibility, effectiveness and sustainability of a community-oriented intervention program. Comparable intervention and control regions were selected in each country. In the intervention regions, a coherent set of intervention modules were implemented, focusing on diet, physical activity and stress-coping capacity. All materials for the interventions were centrally developed and culturally adapted. A detailed description of the IDEFICS intervention program has been recently published [35]. A second survey (T1) reassessed the children 2 years later. The follow-up survey was synchronized with the baseline to account as much as possible for seasonal variation. In fact, the study protocol, closely followed by each survey center, allowed for a 2 year period (±1 month) between T0 and T1.

For the genotyping studies, a randomized age-, sex- and country-matched sample of about 4500 children (4500/8 = 562 from each country), selected according to a minimum set of phenotypes focusing on obesity/overweight, was extracted from the whole IDEFICS population participating in the T0 survey.

Although no formal enquiry about ethnicity was made for privacy and ethical reasons, two questions in the questionnaire filled in by parents or guardians (details on the questionnaires can be found in ref. [36]) were used as proxies for ethnicity. Particularly, place of birth of mother and father and the language habitually spoken at home were used to select only children of white European descent.

The present analysis refers to 4405 children (boys = 2294; girls = 2111), with a complete dataset at T0, including *FTO* rs9939609, BMI and other adiposity measures. 3168 of these children (boys = 1639; girls = 1529) were re-examined two years later (T1) (72% of the T0 sample, control n = 1621, intervention n = 1547).

### Anthropometric Measurements

Children in the IDEFICS surveys underwent a standardized physical examination. Anthropometric data included body weight and height, waist and hip circumferences and the measurement of skinfold thickness. A detailed description of the anthropometric measurements adopted in the IDEFICS study, including intra- and inter-observer reliability, has been recently published [37]. The measurement of weight was carried out using an electronic scale (Tanita BC 420 SMA, Tanita Europe GmbH, Sindelfingen, Germany) to the nearest 0.1 kg with children wearing light clothes and without shoes. Height was measured using a telescopic height-measuring instrument (Seca 225 stadiometer, Birmingham, UK) to the nearest 0.1 cm. BMI was calculated as weight (in kg) divided by height squared (in m).

Waist circumference was measured using an inelastic tape (Seca 200, Birmingham, UK), precision 0.1 cm, range 0±150 cm at the midpoint between the iliac crest and the lower coastal border or 10^th^ rib with the subject in a standing position and recorded at the nearest 0.1 cm. Both triceps and subscapular skinfold thickness ware measured by means of a caliper (Holtain, Holtain Ltd, Pembrokeshire, UK, range 0±40 mm). Measures were taken twice on the right hand side of the body and the mean was calculated.

Waist-to-height ratio was also calculated for further assessment of adiposity. For the definition of overweight/obesity, children were grouped into two categories using the cut-points defined by Cole et al. [38], [39]: 1) underweight (thinness grade III to I) plus normal weight and 2) overweight plus obese.

### Genotyping

Saliva samples were collected from participating children in each country (Oragene DNA Self-Collection Kit, OG-300/OG-250; DNA Genotek Inc., Kanata, Ontario, Canada) and shipped to the central laboratory at the University of Glasgow for DNA extraction. Details on saliva collection and DNA extraction procedures were reported elsewhere [40]. The DNA samples of the children randomly selected for genotyping were sent to the laboratories responsible for genotyping.

The *FTO* rs9939609 genotyping was performed at SNPs Lab of the Institute of Food Sciences, CNR, by a multiplexed end-point assay (TaqMan® Gene Expression Assays, Applied Biosystem, Foster City, CA, USA) to detect variants of a single nucleic acid sequence. The allelic discrimination was made by the 7900HT Fast Real-Time PCR System (Applied Biosystem, Foster City, CA, USA) using standard 384-well reaction plates with standard reagents and standard protocols. Results were analyzed using commercially available software (SDS 2.3, Applied Biosystem, Foster City, CA, USA).

The genotyping success rate was 96.2%. To assess genotyping reproducibility, a random 10% selection of samples was re-genotyped with 100% concordance.

### Statistical Analysis

The Hardy–Weinberg equilibrium genotype distribution in each national sample and in the whole population was analyzed with the χ2 statistics.

Cross-sectional association analysis of *FTO* rs9939609 with anthropometric variables was performed using analysis of variance (GLM General Linear Model) under an additive mode of inheritance with the A allele as risk allele, and adjusting for covariates (age, sex and country of origin).

Prevalence of overweight/obesity at T0 and T1, respectively, and incidence of overweight/obesity over the two year follow-up across the three genotypes were assessed by χ2 statistics. Logistic regression analysis was performed to compare the likelihood of overweight/obesity between the genotypes, adjusting for age, sex and country of origin, with TT as reference category.

Longitudinal analyses were performed using the two-year variation in the outcome variables (T1 minus T0). To test the “genotype X intervention” interaction, we used analysis of variance with genotype, intervention/control group, and “genotype X intervention” factor as the independent variables predicting the change in body mass index and adiposity measures over time. The model was adjusted for sex, age, country of origin, the baseline value for the respective adiposity trait and the individual time interval between T0 and T1. Since no significant “genotype X intervention” interaction was evident, all further analyses were performed adding “intervention/control study group” as covariate in the model.

Logistic regression analysis was performed to compare the likelihood to develop overweight/obesity during the two-year follow-up across the genotypes, adjusting for age, sex, country of origin, intervention/control study group, and the individual time interval between T0 and T1, with TT as reference category.

For the assessment of the possible effect of *FTO* rs9939609 on each individual’s variation of BMI over time, the IDEFICS cohort provided at least 90% power to detect a 20% difference between AA and TT homozygotes at a significance level of *α = *0.05, assuming an additive mode of inheritance.

Statistical analyses were performed using PASW (Predictive Analytics SoftWare) Statistics (version 18; SPSS Inc., Chicago, IL, USA). Results are expressed as means and 95% confidence intervals (CI).

## Results

### Cross-sectional Data

A total of 4405 children were genotyped for rs9939609 in the *FTO* gene. The minor allele frequency (MAF) of rs9939609 was 0.40 in the whole sample in accordance with expectations for population samples of European origin [6]. No significant differences in the genotypic distributions were found between boys and girls (MAF: boys = 0.41; girls = 0.40; p = 0.392). The genotype distribution was in Hardy-Weinberg equilibrium in each national sample and in the whole population, suggesting that the ancestry selection proxies that we used were adequate.

At T0, the A allele of rs9939609 was significantly associated with higher values of BMI (Table 1). The association was also evident for other measures of body fat distribution, including waist circumference (WC), sum of tricipital and subscapular skinfolds (SS) and waist-to-height ratio (W/H) (Table 1).

**Table 1:** Anthropometric variables at T0 according to *FTO* rs9939609 genotypes.

	AA(n = 738)	AT(n = 2086)	TT(n = 1581)	Additivep-value
**Age (years)**	6.07(5.94;6.20)	6.09(6.01;6.17)	6.01(5.92;6.09)	0.37
**BMI (kg/m^2^)**	16.65(16.49;16.81)	16.41(16.31;16.50)	16.18(16.07;16.30)	0.0001
**WC (cm)**	54.93(54.52;55.35)	54.40(54.16;54.65)	53.93(53.65;54.21)	0.0005
**SS (mm)**	19.04(18.52;19.56)	18.41(18.10;18.72)	17.72(17.37;18.07)	0.0002
**W/H**	0.469(0.466;0.472)	0.464(0.462;0.466)	0.462(0.460;0.464)	0.0004

Values are mean (95% confidence interval (CI)).

Multiple regression analysis adjusted for age, sex and country of origin.

BMI = body mass index; WC = waist circumference; SS = sum of skinfolds (subscapular + tricipital); W/H = waist-to-height ratio.

We observed a significant association between the A allele of rs9939609 and the prevalence of overweight/obesity (p = 0.001, chi-square) (Table 2). Logistic regression analysis, applied after adjustment for age, sex and country of origin, revealed a statistically significant association between the A allele and increased risk of overweight/obesity [odds ratio OR_A_ = 1.41, 95% CI = (1.12; 1.77)] as compared with the low-risk T allele homozygotes as reference category. The population attributable risk explained by rs9939609 for overweight/obesity was 11.1%.

**Table 2:** Prevalence of overweight/obesity at T0 and T1 and incidence over the two-year follow-up, according to *FTO* rs9939609 genotypes.

	AA	AT	TT	Additive p-value
**Prevalence at T0 (n)**	22.2% (164)	18.8% (392)	15.9% (252)	0.001
**Prevalence at T1 (n)**	28.7% (155)	21.6% (323)	18.1% (205)	0.00006
**Incidence over the two-year** **follow-up (n)**	11.4% (48)	8.4% (104)	6.1% (58)	0.003

p for χ2 statistics.

### Longitudinal Data

Among the 4405 children examined at T0 and genotyped for *FTO* rs9939609, 3168 children were re-examined two years later (T1) (72% of the T0 sample, control n = 1621, intervention n = 1547). The genotype distribution was equal in the children re-assessed at T1 and in those lost to follow-up (p = 0.95) and in the intervention and control groups (p = 0.93). No difference in the phenotypes distribution between the children re-examined at T1 and those lost at follow-up was observed.

We first analyzed whether there was an interaction between the rs9939609 genotype and the intervention program on the change in the obesity-related traits at 2 years. No statistically significant “genotype X intervention” interaction was found for changes in BMI (F = 0.783, p = 0.457), WC (F = 0.209, p = 0.811), SS (F = 0.973, p = 0.378) and W/H (F = 0.124, p = 0.883), respectively. Therefore, we analyzed the effect of *FTO* rs9939609 adding the intervention/control study group as covariate in the models. Table 3 shows the changes in the anthropometric variables (BMI, WC, SS and W/H) over the two-year follow-up, adjusted for age, sex, country of origin, intervention/control study group, T0 values of the examined variables, and individual time interval between T0 and T1. The number of the copy of A allele of rs9939609 was a statistically significant predictor of the increase in the BMI and other adiposity indices over time, independently of confounders (Table 3).

**Table 3:** Two-year variation in anthropometric variables according to *FTO* rs9939609 genotypes.

	AA(n = 541)	AT(n = 1496)	TT(n = 1131)	Additive p-value
**ΔBMI (kg/m^2^)**	0.960(0.856;1.064)	0.786(0.723;0.848)	0.683(0.611;0.754)	0.0002
**ΔWC (cm)**	4.715(4.384;5.047	4.270(4.071;4.469)	3.907(3.677;4.137)	0.0007
**ΔSS (mm)**	3.327(2.905;3.749)	2.496(2.243;2.748)	2.391(2.096;2.684)	0.002
**ΔW/H**	−0.010(−0.012;−0.007)	−0.013(−0.014;−0.011)	−0.015(−0.017;−0.014)	0.0002

Values are mean (95% confidence interval (CI)).

Multiple regression analysis adjusted for age, sex, country of origin, intervention/control study group, T0 values of the examined variables and individual time interval between T0 and T1.

BMI = body mass index; WC = waist circumference; SS = sum of skinfolds (subscapular + tricipital); W/H = waist-to-height ratio. ΔBMI = (BMI at T1) - (BMI at T0); ΔWC = (WC at T1) - (WC at T0); ΔSS = (SS at T1) - (SS at T0); ΔW/H = (W/H at T1) - (W/H at T0).

Also at T1, we observed a significant association between the A allele of rs9939609 and the prevalence of overweight/obesity (p = 0.00006, chi-square) (Table 2).

Over the two-year follow-up period, 210 new cases of overweight/obesity occurred (8% of the lean children at T0 that were re-examined at T1, control group n = 110, intervention group n = 99) (Table 2). The incidence of overweight/obesity over the two-year follow-up was significantly associated with the A allele (Table 2).

Logistic regression analysis, applied after adjustment for age, sex, country of origin, intervention/control study group, and individual time interval between T0 and T1, revealed that the A allele was associated with a statistically significant increased risk of becoming overweight/obese over the two-year follow-up [OR_A_ = 1.95, 95% CI = (1.29; 2.97)].

## Discussion

In this unique study of a large and well standardized European cohort of children aged 2–9 years, we confirmed that the A allele of *FTO* rs9939609 polymorphism was associated with higher BMI and central body fat distribution and with a greater prevalence of overweight/obesity. Moreover, we showed that this variant was related to a greater increase in body fat and adiposity indices over the two-year follow-up. We did not observe any significant interaction between the *FTO* gene and the community-oriented intervention program implemented as part of the IDEFICS project. Finally, we reported for the first time that the A allele of *FTO* rs9939609 was associated to an increased incidence of overweight/obesity in children.

The cross-sectional analysis of the IDEFICS cohort replicated, in a large and well-characterized population sample of children of European origin, the association of this locus with childhood obesity. The magnitude of the association and the risk for overweight/obesity conferred by *FTO* rs9939609 was comparable with that previously reported in other association studies in children [6], [7], thus confirming this as a childhood obesity susceptibility gene. Of interest, the magnitude of the association is also similar to that observed in adult studies [6], [7], suggesting a relatively stable gene-phenotype association over time for this variant, that may be considered a marker of early onset obesity.

The present study confirms the now established obesity risk A allele, but, more importantly, adds the novel findings obtained by the longitudinal observation of the IDEFICS cohort. In particular, the carriers of the A allele experienced a greater body mass increase and possessed a higher risk of developing overweight/obesity during the period of observation. This finding is intriguing given the relatively short observation period (two years). Other longitudinal studies that explored the genetic influences of *FTO* on the variability of body mass over time have reported heterogeneous findings [13]–[25]. However, most of these studies were retrospective analyses of already established cohorts, with large variation in sample size, often relying on self-reported heights and weights and with vastly different study design. In the present study, the genotyping strategy was part of the study protocol and was agreed before the study commenced, thus allowing the extraction from the whole IDEFICS population of a country-, sex- and age-balanced random sample of children, whose size was *a priori* defined in order to have enough power to detect biologically and clinically relevant differences in the main outcomes.

An additional strength of the present study is the use of precisely standardized phenotypic measurements within the eight European countries participating in the survey. In fact, all measurements were conducted according to detailed standard operation procedures. In particular, subsamples of study subjects were examined repeatedly to calculate the inter- and intra-observer reliability of anthropometric measurements [37].

We also analyzed the effect of the genetic variation in *FTO* on body mass change after the lifestyle intervention. In theory, a lifestyle intervention designed for the primary prevention of obesity might attenuate the risk conferred by the genetic background. Based on preliminary analyses of the effects of the IDEFICS intervention, 16% of the overweight children became normal weight in the intervention region while only 12% in the control region after the two-year intervention. In both regions 9% of the non-overweight became overweight (unpublished data). At the individual level, a significant intervention effect was only observed when looking at BMI in girls (unpublished data). We did not found any interaction between *FTO* and the lifestyle intervention, with the carriers of the risk allele experiencing a significantly greater increase in body mass over time, independently of the study group (control versus intervention). Few studies on relatively small samples previously explored the association between this genotype and the effect of lifestyle intervention programs both in adults [26]–[29] and in children [30]–[32], in general with negative results. Our study on a large and well-characterized sample of European children was in principle powered enough to detect the possible differential effect of the intervention according to the genotype. However, due to the relatively short duration of the intervention and its apparently small effect, we cannot exclude that the early genotyping of *FTO* may be effective in identifying individuals genetically predisposed to obesity that are going to benefit more from a lifestyle intervention than less genetically susceptible individuals. In fact, we cannot rule out the possibility that the mechanism by which *FTO* modulates body mass over time needs a longer period to elicit a differential response to an intervention program.

Despite the many strengths, there are a number of limitations. First, we cannot rule out the possibility that our results may be marginally influenced by population stratification, since no neutral markers were genotyped. Second, the study participants were not nationally representative of the eight European countries involved in the IDEFICS study, as this was never the study objective. We randomly extracted from the whole IDEFICS cohort an age-, sex- and country-matched sample of white European children. In our analysis, however, all data were country-adjusted. Indeed, the adjustment for the eight geographic regions influences the strength of the *FTO* association with BMI only in a minor way (adjusted: p = 0.0002; unadjusted p = 0.0008). Third, although we did not find any interaction between *FTO* and the intervention, the possibility that the intervention program conducted in half of the population could have marginally affected the relationship between *FTO* and the phenotypes of interest over the follow-up cannot be excluded. This possibility was taken into account by adjusting for the control/intervention group in the longitudinal analysis. Once again, this adjustment did not influence the strength of the association between *FTO* and the variation of BMI over the two-year follow-up (adjusted: p = 0.0002; unadjusted p = 0.0002). Finally, the duration of the follow-up was only two year and a 28% loss at follow-up was observed. However, the longitudinal association of *FTO* rs9939606 with adiposity indices clearly emerged even after the relatively short follow-up period and notwithstanding the expected reduction of the sample size, mainly due to the burden of the extensive examination protocol. The planned follow-up of the IDEFICS cohort in subsequent years will allow further evaluation of the association of the *FTO* locus with body mass during the transition from childhood to adolescence.

In summary, we confirmed associations between the *FTO* rs9939609 and higher body mass and overweight/obesity risk in European children. The main finding of the study is that the children with the A risk allele of this variant had a greater increase in body mass and central adiposity over time and therefore were at higher risk of developing overweight/obesity during growth, independently from intervention measures.
